# Sharing Public Health Research Data: Toward the Development of Ethical Data-Sharing Practice in Low- and Middle-Income Settings

**DOI:** 10.1177/1556264615593494

**Published:** 2015-07

**Authors:** Michael Parker, Susan Bull

**Affiliations:** 1 University of Oxford, UK

**Keywords:** biomedical research ethics, data sharing, data release, research data, research governance, low-income countries

## Abstract

It is increasingly recognized that effective and appropriate data sharing requires the development of models of good data-sharing practice capable of taking seriously both the potential benefits to be gained and the importance of ensuring that the rights and interests of participants are respected and that risk of harms is minimized. Calls for the greater sharing of individual-level data from biomedical and public health research are receiving support among researchers and research funders. Despite its potential importance, data sharing presents important ethical, social, and institutional challenges in low-income settings. In this article, we report on qualitative research conducted in five low- and middle-income countries exploring the experiences of key research stakeholders and their views about what constitutes good data-sharing practice.

The sharing of research data is seen as a high priority by researchers and research funders in a wide range of disciplines including mathematics, astronomy, physics, social science, biology, and medicine. In the context of the biosciences, much of the drive toward sharing individual-level biomedical data has its origins in the practices adopted by the Human Genome Project and in the subsequent development of genomics research methods and associated statistical methods for the analysis of large data sets. In genomics, the case for “open access” models of data release was established early on by the Bermuda Principles and the Fort Lauderdale agreement (Human Genome Organisation, 1996, 1997; The Wellcome Trust, 2003). Most large funding bodies now require the depositing of genomic data in a centralized repository as a condition of research funding. From its origins in genomics, data sharing has come to be seen as an important priority across the bioscience landscape and policies mandating the sharing of individual-level data from publicly and privately funded biomedical research are increasingly common, commanding support from large funding bodies, regulatory agencies, journals, and the pharmaceutical industry (European Medicines Agency [EMA], 2014; Godlee & Groves, 2012; Harris, 2011; Medical Research Council, 2011; National Institutes of Health, 2003; Nisen & Rockhold, 2013; Pharmaceutical Research and Manufacturers of America 2013; Research Information Network, 2008; Toronto International Data Release Workshop, 2009; UK Data Archive, 2011; The Wellcome Trust, 2009). Outside the context of academic research, concerns about rigor and transparency in the regulation of pharmaceutical interventions have also seen increased sharing of data from clinical trials (Castellani, 2013; Doshi, Goodman, & Ioannidis, 2013; Eichler, Petavy, Pignatti, & Rasi, 2013; Gotzsche, 2011; Hawkes, 2013; Kirwan, 1997; Nisen & Rockhold, 2013; Rabesandratana, 2013).

Underpinning calls for increased data sharing is a growing recognition of its many potential benefits. These benefits, which are discussed in more detail in our scoping review (Bull, Roberts & Parker, 2015), include potential improvements in the understanding of disease, and in health care to be gained through the greater use of research data. Examples include the generation of novel findings as researchers examine incompletely mined data, apply different hypotheses and analyses to data sets, combine data sets from multiple studies, and develop new research collaborations based on the sharing of data. It is also argued that the sharing of data can improve the reliability of data analysis through its potential to enable the independent evaluation of both the data and the analysis strategies used by researchers, with the potential to identify and reduce inaccuracies or bias in reporting of results. It has also been argued that data sharing may support good governance, allowing for transparency about the uses of public and private funding, increasing researchers’ accountability, fostering increased public faith in research, and lessening unnecessary duplication of research with the attendant costs and burden on participants. Taken together, it is argued, these potential benefits mean that there is a strong public interest in the rapid, effective sharing of research data.

Notwithstanding its claimed benefits, the emergence of data sharing as a requirement of effective research practice in the biosciences and the increasing calls for greater sharing have led to a substantial accompanying literature identifying and analyzing its ethical and social implications. This literature, in both academic and policy domains, has highlighted not only the fact that data sharing presents a range of ethical challenges not previously encountered but also the challenges of taking seriously both ethical arguments for sharing data and those supporting the development of appropriate governance models and mechanisms to ensure the protection of the interests of participants, communities, and the scientists who produce and share data (Abbott, 2014; Antman, 2014; Caulfield et al., 2008; de Vries et al., 2011; Foster & Sharp, 2007; Goldacre et al., 2014; Kuehn, 2014; Lowrance & Collins, 2007; White, 2013; Zarin, 2013). As might be expected, many of the ethical issues relating to data sharing that are discussed in this literature cluster around enduring core concerns in research ethics presented in a new way by developments in data sharing. These include challenges involved in the achievement of valid consent, the nature and scope of responsibilities of researchers to research participants, and the risks and benefits of research. However, in addition to presenting enduring ethical challenges in novel ways, the sharing of health-related data also produces important new problems. These include concerns about the effectiveness of measures to de-identify data, and to protect the privacy of participants (Rathi et al., 2012). Against this background, significant attention has been paid to developing policies and processes for de-identifying such research data over the past three decades (de Wolf et al., 2006; Expert Advisory Group on Data Access [EAGDA], 2013; Hughes, Wells, McSorley, & Freeman, 2014; Office for Civil Rights, 2012; Sieber, 1989). Despite these developments, however, many, including some organizations and advocacy groups that promote data sharing, continue to have privacy-related concerns about the sharing of individual-level data (EMA, 2014; Goldacre, 2013). One way in which such concerns arise most powerfully is in the context of worries about the potential secondary uses of data that are considered “sensitive” or where secondary research will use data to address sensitive or potentially stigmatizing topics (Cooper, 2007; Exeter, Rodgers, & Sabel, 2014; Greenhalgh, 2009; Pearce & Smith, 2011; Sherman & Fetters, 2007). Perhaps unsurprisingly given the concerns outlined above, clinical and public health researchers have been among the slowest to share data (Piwowar, 2011; Tenopir et al., 2011) and in January 2011, recognizing this, major funders of global health research called for increased data sharing to improve public health and set out a shared vision to increase the availability of data generated by their funded research in ways that are equitable, ethical, and efficient (Walport & Brest, 2011).

In addition to concerns about the impact of data sharing on the well-being of participants and the importance of protecting the interests and rights of participants, concerns have also been raised about the potential effects of data sharing on researchers’ career development and about ways to ensure appropriate recognition of those who compile and curate primary data sets (EAGDA, 2014; Manju & Buckley, 2012; Pisani & AbouZahr, 2010; Rani, Bekedam, & Buckley, 2011; Rathi et al., 2012; Walport & Brest, 2011). This is important not only because of the intrinsic importance of fairness in research collaboration but also because of the fact that scientific progress depends upon the promotion of sustainable careers of scientists and scientific capacity more broadly.

Given the concerns mentioned above, it seems likely that significant progress in data sharing will require further work to be done on the development of models of good data-sharing practice capable of commanding the trust and confidence of relevant stakeholders and grounded in shared understandings of what is required for data sharing to be “equitable, ethical and efficient.” These considerations suggest the need for careful consideration of stakeholders’ differing interests in the development of governance policies and processes to carefully judge the balance between the need to share data in a way that maximizes their use, while ensuring that appropriate protections are in place to minimize potential harms (Parker et al., 2009). This also highlights the need for further work on the development and evaluation of models of ethical data sharing capable of garnering public trust and confidence and that of research participants and scientists.

## Sharing Data in Low- and Middle-Income Settings

Given the potential for data-driven research to play a role in addressing the disproportionate disease burden in low and middle settings, increasing emphasis is being placed on the need to promote the sharing of research and public health data generated in such contexts (Manju & Buckley, 2012; Pisani & AbouZahr, 2010; Pisani, Whitworth, Zaba, & AbouZahr, 2010a, 2010b; Rani et al., 2011; Sankoh & Ijsselmuiden, 2011; Tangcharoensathien, Boonperm, & Jongudomsuk, 2010; Walport & Brest, 2011; Whitworth, 2010). In low-income settings where research resources are limited, maximizing the utility of data and minimizing unnecessary duplication of effort are of key importance, and the value of rapidly sharing research data in situations such as public health emergencies to enable timely responses has been recognized (Langat et al., 2011). There is a pressing need for the promotion of high-quality research addressing the diseases affecting the world’s poorest people and it is widely agreed that effective and appropriate data sharing has the potential to play an important role in this. Although this suggests that there are strong ethical arguments in favor of the promotion of efficient data sharing in global health research, care is needed to ensure that data are shared in a way that does not harm vulnerable populations, infringe the rights or interests of those whose data are being utilized, undermine trust in global health research, or threaten the development of sustainable local research capacity (Parker et al., 2009; Pisani et al., 2010a).

Research in low-income settings presents important practical ethical challenges (de Vries et al., 2011), and the practical ethical and governance challenges presented by sharing data from genomic research in low-income settings have been shown to be different in important and morally significant ways to those arising in high-income settings (de Vries & Pepper, 2012; Parker et al., 2009). Identifying, understanding, and addressing these ethical challenges as they arise in diverse—but often interconnected—research settings require empirical social science research to establish a good understanding of the views and perspectives of relevant low- and middle-income stakeholders’ about what constitutes ethical data-sharing practice. Such research, combined with rigorous ethical analysis has the potential to inform the development of models for appropriate and successful research practice including appropriate community engagement processes, consent processes, data-sharing policies, and governance mechanisms for effective data sharing required for the maintaining of confidence and trust in the research process necessary for sustainable research.

Although there have been calls for data-sharing policies and the development of models of good data-sharing practice in research in low-income settings to be informed by stakeholder perspectives, and, where possible, developed by consensus (Manju & Buckley, 2012; Mello et al., 2013; Vallance & Chalmers, 2013; Whitworth, 2010), there are very few empirically grounded accounts of practical and ethical issues arising in the development of data release policies for biomedical and public health research in low-income countries (Bull, Roberts, & Parker, 2015). And, there are no empirically grounded accounts of the views of stakeholders in low- and middle-income settings about the sharing of individual-level data from clinical and public health research (Bull, Roberts, & Parker, 2015). Although some empirical work exploring the ethical aspects of research in developing countries has highlighted data sharing and the sharing of samples as important ethical issues and called for further work (Tindana, Molyneux, Bull, & Parker, 2014), no systematic work has been done in this area.

It is against this backdrop that the articles collected together in this issue report on a multi-site collaborative qualitative research project we conducted in five low-income countries examining stakeholder experiences of, and views about best practices in sharing individual-level data from clinical and public health research. The collection includes a series of six freestanding but connected research articles. In the first article, Bull, Roberts, and Parker (2015), present the findings of a scoping review of the literature relating to data sharing and pertinent research studies. This is followed by individual research articles reporting on the findings of empirical qualitative studies conducted in India, Vietnam, Kenya, Thailand, and South Africa. The methods used for this cluster of related empirical studies are outlined below, and further elaborated in the methods sections of the individual articles.

In their article, Hate et al. report the findings of interviews and focus groups they conducted with public health researchers and research participants at the Society for Nutrition, Education and Health Action (SNEHA), which is a secular non-governmental organization (NGO) working to improve maternal and child health in Mumbai slums (Hate et al., 2015). SNEHA has a team of 190 and works in partnership with both communities and public systems to conduct research and action with a focus on four primary areas: maternal and newborn health, sexual and reproductive health, childhood nutrition, and violence against women and children. As part of this research, data are collected about assets, education, family planning, maternity experience, use of health care providers, agency, mortality and morbidity, nutrition, and violence against women and children.

Merson et al. report on the research they conducted on views about good practice in data sharing at the Oxford University Clinical Research Unit (OUCRU), which is a clinical and public health research unit hosted by the Hospital of Tropical Diseases in Ho Chi Minh City, and the National Hospital for Tropical Diseases in Hanoi (Merson et al., 2015). OUCRU has an integrated clinical science program encompassing patient-orientated clinical research, and aspects of immunology, host and pathogen genetics, molecular biology, virology, epidemiology, and public health. All research is governed by an intricate and multi-level chain of responsibility for every aspect of approval, conduct, and release of information. The article published here reports on the findings of qualitative research with government officers with roles and experience in research and policy development; ethics committee members with research experience and a role in decision making at a major Vietnamese research institution; researchers working in local and international, academic, and commercial institutions; and research participants and their family members.

In their article, Jao et al. report on qualitative research conducted with junior, midcareer, and senior researchers; program health providers and research front-line staff; and, community members, including assistant chiefs and community representatives at the KEMRI-Wellcome Trust Research Programme in Kilifi Kenya (Jao et al., 2015). The KEMRI-Wellcome unit is a multidisciplinary international health research program with more than 750 staff and 70 international scientific collaborations. The program focuses on conducting high-quality research important to health in Africa and includes basic science (parasite and vector biology) and studies on the epidemiology of disease, public health, clinical research, and health systems. Working closely with the Kilifi Ministry of Health, hospital clinical surveillance and population health and demographic data are routinely collected from in-patient wards and the surrounding population of 260,000 people to support health policy and research planning in Kilifi, and health systems and epidemiological research more widely.

Cheah et al. report on interviews and focus groups they conducted with research staff and community members associated with the Wellcome Trust Thailand Major Overseas Programme in Bangkok and in Mae Sot, which is an established collaboration between the Faculty of Tropical Medicine, Mahidol University, the University of Oxford, and the Wellcome Trust (Cheah et al., 2015). Primary research interests at the program are the epidemiology, diagnosis, pathophysiology, pharmacology, and treatment of infectious diseases throughout Asian and other low- and middle-income settings. The unit currently has 60 to 70 active clinical studies on malaria and other tropical diseases such as meliodosis.

In their article, Denny, Silaigwana, Wassenaar, Bull, & Parker, report on qualitative research with senior researchers with personal experience of data sharing and junior research and community stakeholders drawn from three large South African research centers that regularly collect, store, and share data in their respective capacities (Denny, Silaigwana, Wassenaar, Bull, & Parker, 2015). This included two primarily biomedical research organizations—one a low-risk institute that is primarily engaged in fundamental biomedical research and specimen collection and the other a large health research and clinical trials unit, focused on HIV, TB, and AIDS prevention research, and a large research organization that conducts social scientific research for both NGOs and international development agencies.

Finally, these setting-specific articles are followed by an overarching article in which Bull, Cheah et al., (2015) explore the implications of the analyses of stakeholder views reported in the individual articles for developing contextually appropriate models of best practice in ethical data-sharing practices in low- and middle-income settings. The article reviews the findings of the multi-sited research project as a whole and goes on to make some recommendations about ways forward in the development of models of good practice for data sharing in low-income settings including suggestions for further research.

## Method

The methodological starting point for this multi-site study was the need to develop a robust but appropriately pragmatic model for the exploration of a core, shared set of research questions at five diverse but complementary research settings using data-collection and analysis procedures developed for use across the project as a whole. Our aim was to ensure that the research at each site was a rigorous stand-alone project that was appropriately responsive to the local context and at the same time capable of supporting cross-site comparisons of findings from these very different settings, which despite their differences also had much in common because of their involvement in medical and public health research (Herriott & Firestone, 1983).

Qualitative studies were conducted simultaneously in India, Kenya, South Africa, Thailand, and Vietnam. The selected settings had long-standing institutions conducting various forms of medical and public health research, were based in a variety of socio-economic contexts, and each had a strong interest in and available local expertise to examine stakeholder views on data sharing.

To ensure that the development of a study was both cohesive and responsive to specific contexts, three face-to-face meetings and fortnightly teleconferences took place during the 18-month study to provide frequent interaction, to ensure that identification of core topics, data-collection methods, and analysis frameworks were developed collaboratively. Regular communication made it possible for methodological and other aspects of the research to be discussed by the team as a whole throughout the life of the project in tandem with data collection and analysis. At an initial face-to-face planning meeting in June 2013, researchers agreed to use semi-structured interviews and focus groups to explore key stakeholders’ experiences, understandings of sharing individual-level research data, concerns about future data use, and views about best practices in data sharing. Drawing on an initial analysis of the relevant literature and on the extensive practical experience of the research partners, a list of 19 potential topics to be addressed during data collection was compiled. Agreement was also reached about a core set of types of stakeholder who were to be engaged at each site comprising senior researchers and study principal investigators, junior researchers and staff recruiting participants, and members of communities from which research participants were drawn. To the degree that this was possible, attempts would also be made to engage with additional groups of key stakeholders of relevance at specific sites, including participants in medical or public health research, members of community advisory boards, bioethicists, data managers, and research managers.

Although there is a published literature supporting the use of deliberative discussions to engage stakeholders about ethical issues relating to research as a research method, this literature also acknowledges that such methods present a number of challenges, particularly where complex, unfamiliar, and technical issues are addressed (Haga & O’Daniel, 2011; Kim, Wall, Stanczyk, & De Vries, 2009; Marsh, Kamuya, Rowa, Gikonyo, & Molyneux, 2008; Molster et al., 2013; Parker, 2007). Following discussion of these issues and of the previous experience of qualitative research at each of the settings, it was decided that interviews would be an appropriate means of data collection for stakeholders with some experience of working with and sharing research data. For stakeholders with less familiarity with data sharing including junior research staff community members and research participants, focus groups discussions structured around the discussion of vignettes would be adopted. Three potential vignettes, based on examples of data collection and sharing at the research sites, were developed and discussed. It was hoped that the vignettes would provide both a structure for information provision about the kinds of data that were or could be valuable to share, and act as the basis for structured probes on ethically relevant issues in the group discussions. It was envisaged that probes would aim to identify perceived advantages and concerns about data sharing, and views about how data should be shared, and any restrictions that might be appropriate. Additional probes would seek to determine if views changed depending on factors such as with whom and where data were shared, what kinds of secondary use might be made of the data, and what kinds of data were shared. Where participants’ views changed in response to probes, they would be encouraged to reflect on potential reasons for such changes and the implications of these for best practices in data sharing. Examples of the materials used in data collection for this study are described in greater detail in the individual articles reporting on the results from each study setting (Cheah et al., 2015; Denny, Silaigwana, Wassenaar, Bull, & Parker, 2015; Hate et al., 2015; Jao et al., 2015; Merson et al., 2015).

Following the initial planning workshop, each of the project partners liaised with their own research groups to develop locally appropriate study protocols and data-collection methods on the basis of the broad approach agreed. During a subsequent face-to-face meeting 5 months later, in November 2013, a further modified set of topics to be addressed in interviews was developed, drawing on the materials developed at individual sites and further work was done on all aspects of the research design. A core group of topics emerging from the literature and topic guides were identified, and represented as a set of nodes to provide a framework for analysis at each of the sites. Further development also took place of means of developing explanatory scenarios for focus groups and structured probes to facilitate consideration and discussion of complex and unfamiliar topics.

Subsequent to these discussions, and the obtaining of ethics approval, data collection for the study took place from January 2014 to October 2014. During data collection and iterative analysis, online discussions and sharing of study materials took place on a secure University of Oxford Nexus Sharepoint website established for the study. Preliminary findings and themes emerging from the data at each site were discussed in tandem with data collection, which informed the further refinement of data-collection materials. A sample of transcripts from four of the sites were cross-coded by researchers from multiple sites to evaluate how the pre-specified deductive nodes were being used in coding, the value of the nodes, and how they might be complemented by inductive grounded nodes developed at each of the sites (Ezzy, 2002).

A 4-day face-to-face research meeting in July 2014 provided an opportunity for the research teams to have a more extensive face-to-face discussion of their data collection and analysis. At this meeting, similarities and differences in stakeholder views within and between sites were reviewed, and informed reflections on the development of grounded descriptive codes at each of the sites and development of thematic codes relevant to one or more sites. Framework analysis approaches to charting themes emerging from the data at each of the sites were collaboratively discussed, and informed subsequent analysis of the data sets for each site (Green & Thorogood, 2007; Smith & Firth, 2011). Subsequent to the meeting, research teams at each site completed their analysis, and draft papers were circulated for discussion prior to submission.

## Concluding Comments

The articles collected in this special issue report on a multi-sited qualitative research project conducted in India, Thailand, Kenya, Vietnam, and South Africa to better understand the views of key stakeholders about the key requirements for good data-sharing practice capable of garnering well-founded trust and confidence of participants and researchers in low-income settings. The research was conducted against the background of increasing international calls for greater sharing of research and public health data from low-income settings and claims about the benefits of such data sharing for the understanding of and development of interventions in diseases affecting the world’s poorest people. To our knowledge, this is the first study to explore these issues in depth and is also the first study to attempt to do so using a multi-sited approach that reflects the collaborative nature of global health research itself. It is our hope that this study will play a role in informing the development of models of good, ethical data-sharing practice and also prompt further research on this important set of topics (Bull, Cheah, et al., 2015).
